# Vascular calcification in patients with type 2 diabetes: the involvement of matrix Gla protein

**DOI:** 10.1186/1475-2840-13-85

**Published:** 2014-04-24

**Authors:** Sophie Liabeuf, Bourron Olivier, Cees Vemeer, Elke Theuwissen, Elke Magdeleyns, Carole Elodie Aubert, Michel Brazier, Romuald Mentaverri, Agnes Hartemann, Ziad A Massy

**Affiliations:** 1 INSERM U1088, Jules Verne University of Picardy, F-80000 Amiens, France; 2 Clinical Research Centre, Division of Clinical Pharmacology, Amiens University Hospital, Jules Verne University of Picardy, F-80000 Amiens, France; 3 Diabetology Department, AP-HP, Pitie-Salpétrière Hospital and Pierre, Marie Curie University of Paris, F-75005 Paris, France; 4 VitaK, Maastricht University, Maastricht, Netherlands; 5 Division of Nephrology, Ambroise Paré Hospital, Paris-Ile-de-France-Ouest University (UVSQ), 9 avenue Charles de Gaulle, F-92104 Boulogne-Billancourt, France

**Keywords:** Matrix gla protein, Type 2 diabetes, Peripheral calcification

## Abstract

**Background:**

Matrix Gla protein (MGP) is an important inhibitor of calcification. The objective of the present study of patients with type 2 diabetes and normal or slightly altered kidney function was to evaluate levels of inactive, dephospho-uncarboxylated MGP(dp-ucMGP) and total uncarboxylated MGP(t-ucMGP) and assess their links with biological and clinical parameters (including peripheral vascular calcification).

**Methods:**

The DIACART study is a cross-sectional cohort study of 198 patients with type 2 diabetes and normal or slightly altered kidney function. Matrix Gla protein levels were measured with an ELISA and all patients underwent multislice spiral computed tomography scans to score below-knee arterial calcification.

**Results:**

In the study population as a whole, the mean dp-ucMGP and t-ucMGP levels were 627 ± 451 pM and 4868 ± 1613 nM, respectively. Glomerular filtration rate, age and current vitamin K antagonist use were independently associated with dp-ucMGP levels. When the study population was divided according to the median peripheral arterial calcification score, patients with the higher score displayed significantly lower t-ucMGP and significantly higher dp-ucMGP levels. Furthermore, plasma dp-ucMGP was positively associated with the peripheral arterial calcification score (independently of age, gender, previous cardiovascular disease and t-ucMGP levels).

**Conclusions:**

High dp-ucMGP levels were independently associated with below-knee arterial calcification score in patients with type 2 diabetes and normal or slightly altered kidney function. The reversibility of the elevation of dp-ucMGP levels and the latter’s relationship with clinical events merit further investigation.

## Background

Peripheral arterial disease (PAD) is a major vascular complication and the leading cause of amputation in people with diabetes. In patients with PAD, the tibial artery calcification score is a useful tool for identifying patients at high risk of amputation, since the score has greater predictive value than traditional risk factors [1]. Indeed, diabetes accelerates atherosclerosis and increases the incidence of vascular calcification (VC) [2,3]. In people with diabetes, VC is present in both coronary arteries and arteries of the lower limbs. Furthermore, VC is an independent predictor of cardiovascular and overall mortalities in patients with type 2 diabetes [4]. Various epidemiologic studies have identified specific biomarkers (including osteoprotegerin, osteocalcin and others) for VC in this population [5,6].

**Figure 1:**
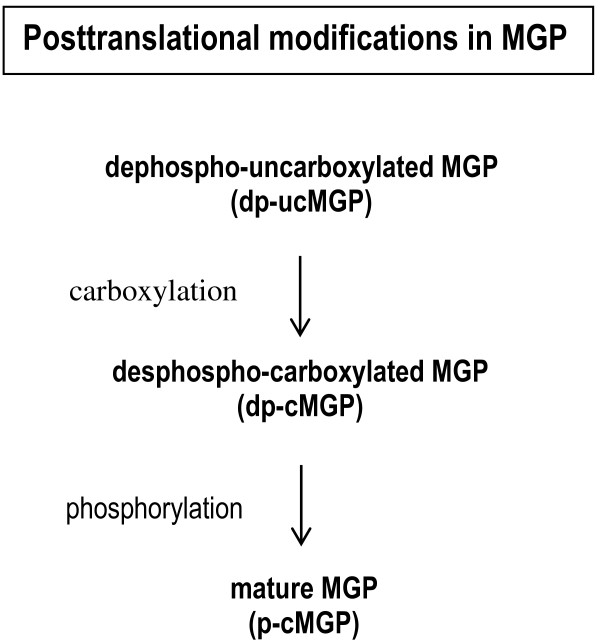
**The different forms of the matrix Gla protein (MGP).** legend: MGP needs two posttranslational modifications for maturation: glutamate carboxylation and serine phosphorylation. Both modifications are only partially accomplished. Besides the non-modified form (dp-ucMGP) also partially modified species (dp-cMGP and p-ucMGP) and the fully maturated form p-cMGP are present in the circulation. In this paper we have tested dp-ucMGP and total ucMGP (t-ucMGP) (which consists of the sum of dp-ucMGP and p-ucMGP). Since the plasma concentration of p-ucMGP is about 10 thousand fold higher than that of dp-ucMGP, the t-ucMGP assay virtually measures p-ucMGP.

One of the most interesting calcification inhibitors is matrix Gla-protein (MGP), a vitamin K-dependent protein that is expressed by smooth muscle cells, fibroblasts, chondrocytes and endothelial cells in a variety of tissues (including arterial vessel wall). There is evidence to suggest that full activation of MGP requires posttranslational carboxylation and phosphorylation (Figure 1) [7,8]. In order to measure vitamin K status, assays were developed to measure the conformations of MGP with the least activity, i.e. forms with no posttranslational modifications (dephospho-uncarboxylated MGP (dp-ucMGP)) or at least no gammaglutamyl carboxylation (total uncarboxylated MGP (t-ucMGP)) [9]. In fact, dp-ucMGP does not possess calcium-binding groups and is not retained in the vessel wall. Hence, the dp-ucMGP assay is a direct marker of vascular vitamin K status. In both healthy subjects and patients, poor vascular vitamin K status (corresponding to high circulating dp-ucMGP levels) is regarded as a risk marker for forthcoming arterial calcification. Indeed, dp-ucMGP was found to be associated with the severity of aortic calcification in patients with chronic kidney disease (CKD) [10]. Circulating t-ucMGP levels are at least 1000-fold higher than those of dp-ucMGP, and are thought to consist mainly of phosphorylated uncarboxylated MGP (p-ucMGP) species, i.e. MGP-related antigens with between 1 and 3 high-affinity calcium-binding groups. This explains why immunohistochemical techniques invariably find ucMGP to be closely associated with calcium deposits in the vasculature; in turn, this observation is consistent with the inverse association between circulating t-ucMGP levels and the VC score.

A few studies have evaluated the association between MGP levels and VC in patients with diabetes [11-13], although the researchers used different antibodies to determine either circulating dp-ucMGP [11], t- ucMGP [12] or other conformations of ucMGP [12,14]. There are few data on (i) the levels of different MGP forms in a selected population with type 2 diabetes and (ii) the relationships between these various forms and peripheral artery calcification. This knowledge would be useful, since as Dalmeijer et al. have recently demonstrated that high dp-ucMGP levels were associated with increased cardiovascular risk (PAD and heart failure) in patients with type 2 diabetes [11]. Furthermore, Doyon et al. used a rat model of diabetes to show that the decrease in active carboxylated MGP (cMGP) levels could be due to an impairment of gammaglutamate carboxylation - suggesting that the signalling pathways involved in gammaglutamate carboxylase regulation are altered in diabetes [15].

The objective of the present study of patients with type 2 diabetes and normal or slightly altered kidney function was to evaluate (i) t-ucMGP and dp-ucMGP levels, (ii) biochemical and clinical parameters associated with differences in dp-ucMGP and t-ucMGP levels and (iii) the potential association between MGP levels and peripheral VC.

## Materials and methods

### Ethics statement

The study was performed in accordance with the principles of the Declaration of Helsinki and in compliance with the International Conference on Harmonization's guidelines on good clinical practice. The study protocol was approved by the local independent ethics committee (*Comité de Protection des Personnes*, Paris, France) prior to the initiation of any study-specific procedures. All patients were provided with full information on the study objectives and procedures and gave their written informed consent to participation.

### Patient selection

In the "Diabète et Calcification Arterielle" (DIACART) cross-sectional study, 198 patients with type 2 diabetes from the Diabetology Department and the Cardiology Department at Pitié-Salpêtrière Hospital (Paris, France) were included over an 8-month period. The objective of DIACART study was to gain a better understanding of the pathophysiology of peripheral artery calcification in diabetic patients. The main inclusion criteria were (i) type 2 diabetes, with at least coronary artery disease and/or peripheral arterial occlusive disease and (ii) age >50 for men and >60 for women. The main exclusion criteria were (i) an estimated glomerular filtration rate (eGFR, calculated with the Modification of Diet in Renal Disease equation) <30 ml/min and (ii) a history of lower limb angioplasty and/or bypass.

### Study protocol

All patients were hospitalized for the day in order to perform clinical evaluations, laboratory blood tests and a multislice spiral computed tomography (CT) scan. A patient interview focused on comorbidities and the personal disease history. The patient’s medical records were reviewed to check the information and to record vitamin K antagonist (VKA) use.

Previous CVD was defined as a history of any of the following events: myocardial infarction, stroke or any surgical procedures for angina or coronary disease (including percutaneous transluminal angioplasty).

### Laboratory tests

Blood and urine samples were collected after an overnight fast for measurement of routine biochemistry, glycaemia, HbA1C, high-sensitivity C-reactive protein, calcium, phosphorus, 25-OH vitamin D, intact parathyroid hormone (PTH), triglycerides and cholesterol.

Selected assays (including dp-ucMGP and t-ucMGP assays) were performed after the samples had been frozen, stored at -80°C and thawed. A dual-antibody ELISA was used to measure dp-ucMGP levels; the capture antibody was directed against the non-phosphorylated MGP sequence 3–15 (mAb-dpMGP; VitaK BV, Maastricht, The Netherlands) and the detecting antibody was directed against the uncarboxylated MGP sequence 35–49 (mAb-ucMGP; VitaK BV). The same antibodies have already been used for immunohistochemical staining [16-18]. Intra-assay variability was 5,6% for dp-ucMGP and 8,9% for t-ucMGP, when inter-assay variability was 9,9% for dp-ucMGP and 11,4% for t-ucMGP. In 81 age-matched controls, the mean level of dp-ucMGP was 557 ± 277 pM (median: 522 pM) (measured separately in archived samples).

A competitive (single-antibody) ELISA was used to measure t-ucMGP levels, as described previously [9,19]. In 81 age-matched controls, the mean t-ucMGP level was 4282 ± 1100 nM (median: 4109 nM).

### Imaging for calculation of the below-knee arterial calcification score

Tibial artery calcium scoring was performed after scanning with a 128-slice multidetector CT scanner (Somatom Definition Flash, Siemens Healthcare, Forchheim, Germany) in the craniocaudal direction, from the bottom of the patella down to the ankle region. Contrast agent was not used. Cross-sectional slices (with: 3 mm) were analyzed individually. The analysis was performed with a commercially available software package (Heartbeat CaScore, Philips Healthcare, Eindhoven, The Netherlands) by radiologists who were not aware of the results of the clinical examination or laboratory assays, On cross-sectional images, areas of calcification along below-knee arteries with a density ≥130 Hounsfield units and a surface >1 mm^2^ were identified automatically. The calcification scores (determined according to the method described by Agatston et al. [20]) for each of the main below-knee arteries (the distal popliteal, anterior tibial, posterior tibial and peroneal arteries) were summed to obtain the overall calcification score.

### Statistical analyses

Data were expressed as the mean ± SD, median or frequency, as appropriate. The study patients were stratified according to the median dp-ucMGP level. This median value appears to be the best available cut off, according to the receiver operating characteristic curve for VC (with a sensitivity of 0.65 and a specificity of 0.70). Intergroup comparisons were made using a χ^2^ test for categorical variables and Student’s t test or the Kruskall-Wallis test for continuous variables. Spearman correlations were used to identify parameters correlated with dp-ucMGP levels and t-ucMGP. For parameters presenting a non-Gaussian distribution, log-normalized values were considered in tests that require normally distributed variables. Multiple linear regression analysis was used to select factors that were independently associated with dp-ucMGP levels. Unadjusted (Table 1) and adjusted (Table 2) logistic regression analyses were performed to evaluate the association between the peripheral calcification score (as categorized by the median) and dp-ucMGP. A p value ≤ 0.05 was considered to be statistically significant. All statistical analyses were performed using SPSS software (version 13.0, SPSS Inc., Chicago IL, USA) for Windows (Microsoft Corp., Redmond WA, USA).

**Table 1:** Univariate logistic regression analysis: variables associated with calcification score divided by the median (n = 198 patients)

	**Odds ratio (95% confidence interval)**	**p**
Age	1.07 (1.03; 1.11)	<0.0001
Ln [t-ucMGP]	0.27 (0.11; 0.63)	0.003
Ln [dp-ucMGP]	1.88 (1.21; 2.91)	0.005
Gender	2.56 (1.36; 4.75)	0.006
Previous CVD	2.56 (1.36; 4.75)	0.004

For abbreviations, please refer to Table 3.

Variables not significantly associated: diabetes duration, smoking status, VKA treatment, body mass index, SBP, DBP, GFR-MDRD, glycaemia, HbA1c, microalbuminuria, IL6, cholesterol, C-reactive protein, 25(OH) Vit D, calcium, phosphate.

**Table 2:** Multivariate logistic regression analysis: variables independently associated with the calcification score divided by the median (n = 198 patients)

	**Odds ratio (95%confidence interval)**	**p**
Age per 1 year	**1.06 (1.02; 1.11)**	**0.006**
Ln [t-ucMGP]	0.44 (0.17; 1.15)	0.092
Ln [dp-ucMGP]	**1.88 (1.14; 3.11)**	**0.014**
Male gender	**3.84 (1.66; 8.88)**	**0.002**
Previous CVD	**2.56 (1.20; 4.85)**	**0.013**

Variables entered into the model: age, Ln [dp-ucMGP], Ln [t-ucMGP], gender, previous CVD.

## Results

### Baseline characteristics

Table 3 presents the main clinical and biochemical characteristics for the entire cohort and for the subgroups above and below the median dp-ucMGP (559.5 pM) value. Patients with higher plasma dp-ucMGP levels were significantly older, were more likely to taking VKAs and had higher body mass index, PTH levels and triglyceride levels and a lower eGFR. Patients with higher plasma t-ucMGP levels (i.e. above to the median t-ucMGP value (4741 nM) were significantly younger, had higher body mass index, diastolic blood pressure, calcium and triglyceride levels and lower PTH levels. It is noteworthy that although only six patients were being treated with VKA, their dp-ucMGP levels were much higher than in untreated patients (the mean and median dp-ucMGP levels were 2093 ± 1125 pM and 1984 pM for VKA-treated patients and 581 ± 327 pM and 553 pM for non-VKA-treated patients). Treatment with renin angiotensin aldosterone inhibitors was not associated with changes in levels of MGP forms or in the calcification score. We also compared patients as a function of their HbA1c levels (above vs. below 7%) but found no difference in terms of either VC, t-ucMGP or dp-ucMGP.

**Table 3:** Baseline characteristics as a function of the median plasma dp-ucMGP level

	**All**	**[dp-ucMGP] ≤ 559.5 pM**	**[dp-ucMGP] > 559.5 pM**	**p**
	**n = 198**	**n = 99**	**n = 99**	
**Age (years)**	**64 ± 8**	**63 ± 9**	**66 ± 8**	**0.002**
Diabetes duration (years)	15 ± 10	14 ± 10	15 ± 9	0.358
Male gender n (%)	158 (80)	82 (83)	76 (77)	0.288
**Body mass index (kg/m**^**2**^**)**	**29 ± 5**	**28 ± 5**	**30 ± 5**	**<0.0001**
SBP (mmHg)	127 ± 17	126 ± 17	128 ± 17	0.377
DBP (mmHg)	73 ± 9	73 ± 9	72 ± 8	0.487
Smoking habit n (%)	119 (60)	58 (59)	61 (62)	0.663
Previous CVD n (%)	139 (70)	66 (67)	73 (74)	0.277
Glycaemia (mmol/l)	8.2 ± 2.8	8.0 ± 2.6	8.3 ± 2.9	0.401
	(7.8)	(7.6)	(8.0)	
HbA1c (%)	7.8 ± 1.5 (7.5)	7.7 ± 1.4	7.9 ± 1.5	0.572
		(7.5)	(7.5)	
**GFR MDRD (mmol/l)**	**80 ± 19**	**82 ± 19**	**70 ± 19**	**<0.0001**
**Microalbuminuria (mg/l)**	**166 ± 840**	**169 ± 1132**	**162 ± 373**	**<0.0001**
	**(23)**	**(14)**	**(33)**	
Calcium (mmol/l)	2.30 ± 0.10	2.30 ± 0.10	2.34 ± 0.10	0.055
Phosphate (mmol/l)	1.02 ± 0.15	1.04 ± 0.20	1.00 ± 0.20	0.164
**Intact PTH**	**54.5 ± 27.5**	**50.8 ± 26.0**	**58.5 ± 28.9**	**0.030**
**(pg/mL)**	**(46.9)**	**(45.2)**	**(52.0)**	
25(OH)Vit D (ng/ml)	13.8 ± 8.4	14.4 ± 8.2	13.2 ± 8.5	0.175
	(12.0)	(13.0)	(11.0)	
CRP	2.2 ± 1.5	2.2 ± 2.6	2.2 ± 2.5	0.725
(mg/l)	(1.2)	(1.3)	(1.1)	
**Triglycerides**	**1.6 ± 1.1**	**1.4 ± 1.1**	**1.6 ± 0.9**	**0.039**
**(mmol/L)**	**(1.3)**	**(1.2)**	**(1.4)**	
Total cholesterol (mmol/l)	3.7 ± 0.9	3.7 ± 0.8	3.8 ± 0.9	0.283
LDL cholesterol	1.9 ± 0.7	1.9 ± 0.7	2.0 ± 0.8	0.403
(mmol/L)	(1.8)	(1.8)	(1.8)	
Total cholesterol/HDL	3.7 ± 1.6	3.5 ± 1.1	3.9 ± 1.8	0.053
cholesterol	(3.5)	(3.3)	(3.5)	
**Peripheral calcification score**	**2528 ± 5779**	**1609 ± 3983**	**3447 ± 7040**	**<0.0001**
	**(524)**	**(274)**	**(1096)**	
t-ucMGP (nM)	4868 ± 1613	4815 ± 1689	4921 ± 1539 (4787)	0.460
	(4741)	(4616)		
dp-ucMGP (pM)	627 ± 451	342 ± 142	912 ± 474	NA
	(559)	(352)	(755)	
**VKA treatment n (%)**	**6 (3)**	**0 (0)**	**6 (6)**	**0.013**

Data are given as means ± SD for normally distributed measures with addition of (median) for non-normally distributed values for variables with a non-Gaussian distribution or as the number (percentage) for binary variables.

SBP: systolic blood pressure; DBP: diastolic blood pressure; CVD: cardiovascular disease; HbA_1c_: haemoglobin A_1C_; GFR MDRD: Glomerular filtration rate calculated with the Modification of Diet in Renal Disease formula; PTH: parathyroid hormone; CRP: C-reactive protein, t-ucMGP: total uncarboxylated matrix Gla-protein; dp-ucMGP: dephospho-uncarboxylated matrix Gla-protein. VKA: vitamin K antagonist.

### MGP values as a function of the calcification score

Patients with an above-median peripheral arterial score had significantly lower t-ucMGP levels (median: 4941 nM vs. 4550 nM, respectively; p = 0.006) and significantly higher dp-ucMGP levels (median: 480 pM vs. 652 pM respectively; p = 0.001).

### Associations between dp-ucMGP and calcification

A univariate correlation analysis with plasma dp-ucMGP essentially confirmed the results as described in Table 3 (Table 4). These findings did not change when the six VKA-treated patients were excluded from the analyses. Moreover, a multivariate linear regression analysis (including all variables significantly associated with plasma dp-ucMGP levels in the univariate analyses) identified eGFR (p < 0.0001), age (p =0.002) and current antivitamin K use (p < 0.0001) as independent factors.

**Table 4:** Univariate correlations: variables associated with plasma dp-ucMGP levels and t-ucMGP levels

	**dp-ucMGP levels**		**t-ucMGP levels**	
	**r**	**p**	**r**	**p**
**Age**	**0.294**	**<0.0001**	**-0.306**	**<0.001**
Diabetes duration	0.077	0.278	-0.133	0.062
**Body mass index**	**0.250**	**<0.0001**	**0.291**	**<0.0001**
SBP	0.027	0.710	**0.171**	**0.016**
DBP	-0.105	0.142	0.324	**<0.0001**
Glycaemia	0.046	0.520	0.130	0.069
HbA1c	0.034	0.638	0.105	0.141
**GFR MDRD**	**-0.410**	**<0.0001**	**0.211**	**0.003**
**Microalbuminuria**	**0.307**	**<0.0001**	-0.021	0.766
**Calcium**	**0.145**	**0.042**	**0.255**	**<0.0001**
Phosphate	-0.089	0.214	-0.095	0.182
**Intact PTH (pg/mL)**	**0.197**	**0.005**	**-0.256**	**<0.0001**
25(OH)D	-0.095	0.181	-0.009	0.903
C-reactive protein	0.137	0.055	0.110	0.120
**Triglycerides**	**0.215**	**0.002**	**0.312**	**<0.0001**
Total cholesterol	0.084	0.240	0.079	0.268
**Total cholesterol/HDL cholesterol**	**0.188**	**0.008**	**0.202**	**0.004**
t-ucMGP	0.052	0.467	1.000	**<0.0001**
**Peripheral calcification score**	**0.260**	**<0.0001**	**-0.272**	**<0.0001**

For abbreviations, please refer to Table 3. r = Spearman correlation coefficient.

In the present cohort, we found that age, male gender, previous CVD, and dp-ucMGP levels were positive risk factors for an elevated calcification score, whereas t-ucMGP appeared to protect against VC in a univariate analysis (Table 1). Furthermore, in a multivariate logistic analysis, dp-ucMGP appeared to an independent predictor of peripheral arterial calcification (independently of age, gender, previous CVD and t-ucMGP levels) (Table 2). Patients with high dp-ucMGP presented an elevated risk of VC, independently of classical risk factors. Similar evidence was obtained after the exclusion of VKA-treated patients from the analysis.

## Discussion

The present study is the first to show that in patients with type 2 diabetes and normal or slightly altered kidney function, dp-ucMGP levels (a marker for vitamin K status) are independently associated with age, eGFR and VKA treatment. More importantly, peripheral VC is associated with dp-ucMGP (independently of age, gender, previous CVD and t-ucMGP levels).

Matrix Gla protein is a strong inhibitor of VC, as revealed by the development of massive VC in MGP knockout mice [21]. Indeed, MGP is a vitamin K-dependent inhibitor of calcium phosphate precipitation and crystal formation in the vessel wall [22]. Furthermore, it suppresses the activity of bone morphogenetic proteins 2 and 4 [22,23]. The value of MGP as a calcification biomarker has been evaluated in various cohorts, and a number of assays have been developed to measure its various conformations. In the present study, we evaluated circulating t-ucMGP and dp-ucMGP concentrations. We focused on dp-ucMGP because this form has low affinity for vascular calcium deposits and is secreted into the bloodstream by vascular smooth muscle cells. Moreover, dp-ucMGP is a well-known marker for vascular vitamin K status. High levels of dp-ucMGP have been associated with aortic calcification (independently of classical risk factors) in patients at different stages of CKD [10,24]. Similarly, the present study is the first to demonstrate that in patients with type 2 diabetes and normal or slightly altered kidney function, dp-ucMGP levels were positively associated with peripheral artery calcification (independently of age, gender, previous CVD and t-ucMGP). It is important to study VC in diabetic patients, since as calcification process could present specific features. Indeed Flammer et al. recently reported that patients with elevated HbA1c levels had a significantly higher percentage of circulating blood mononuclear cells expressing the osteoblastic marker osteocalcin. However, further research is required to establish whether these cells increase VC [25]. On the same lines, a distinct subpopulation of circulating cells expressing osteocalcin and bone alkaline phosphatase had procalcific activity in type 2 diabetic patients [26].

Indeed, the present study is the first to have evaluated the potential role of this important calcification inhibitor and its relationship with peripheral artery calcification (as evaluated with a CT-based methodology). This result is in agreement with recently published data from a prospective study of 518 patients with type 2 diabetes (mean follow-up period: 11.2 years); Dalmeijer et al. demonstrated a strong, independent relationship between high dp-ucMGP and cardiovascular risk (and PAD in particular), whereas t-ucMGP levels were not associated with CVD [11]. Indeed, the presence of medial calcification (which is particularly present in peripheral arteries [27]) could explain the positive correlation between dp-ucMGP levels and PAD.

Only one study (in outpatients with stable CVD) reported that higher t-ucMGP levels are associated with lower mitral annular calcification (MAC) in persons without diabetes and higher MAC in persons with diabetes [12]. In the present study, a univariate analysis revealed an inverse relationship between t-ucMGP and peripheral artery calcification; however, this link was no longer significant after adjustment for dp-ucMGP. The conflicting results in these two studies might be due to differences in the calcification site evaluated (MAC versus peripheral calcification) and patient recruitment (outpatients with stable CVD versus patients in a diabetes ward). However, it appears to be important to assay for dp-ucMGP when focusing on peripheral VC, since this factor is still associated with peripheral artery calcification after multiple adjustments. Furthermore, Dalmeijer et al. reported that dp-ucMGP levels (but not t-ucMGP levels) were associated with PAD. These findings suggest that VC sites differ in terms of their specific features and biomarkers. This is of particular interest in the pathophysiology of VC in patients with diabetes, who are particularly prone to this condition. Indeed, peripheral artery calcification is very frequent in patients with diabetes and can lead to amputation.

Furthermore, we identified eGFR, age and current VKA use as independent factors for dp-ucMGP levels. It is already know that in patients with CKD, levels of inactive forms of MGP increase progressively [10]. Even when patients with normal or slightly altered kidney function are selected, eGFR is still a strong predictor of MGP levels. It is noteworthy that the link between dp-ucMGP levels and calcification appeared to be independent of kidney function in the present study.

The use of VKAs appears to be an important predictor of dp-ucMGP; the six VKA-treated patients had dp-ucMGP levels that were around 4 times greater than in the other patients. Indeed, VKA treatment indirectly inhibits the carboxylation of vitamin K-dependent proteins via interactions with vitamin K epoxide reductase. Hence, VKA treatment inhibits the gamma-carboxylation of MGP and leads to an increase in inactive forms of MGP and thus prompts VC [28,29]. Indeed, epidemiological studies performed in the last decade have revealed VC in warfarin-treated patients [29].

Until recently, VKAs were the only drugs for long-term treatment of thromboembolic disorders. However, novel new oral anticoagulant agents (NOACs) have now emerged (e.g. factor Xa inhibitors such as rivaroxaban, apixaban and factor IIa inhibitors such as dabigatran). Unlike VKAs, the NOACs do not interfere with vitamin K-dependent proteins and may thus be safer with regard to VC. This advantage is particular important in diabetic populations, who are particular prone to the development of VC. However, this hypothesis needs to be confirmed in prospective trials and balanced against the efficacy and safety of NOACs.

Given that VC in general and peripheral calcification in particular are major problems in patients with diabetes, modulation of vitamin K status might be an interesting therapeutic option. Indeed, vitamin K is gaining increasing attention in terms of its therapeutic potential in VC [30,31]. In a recent pilot study, vitamin K supplementation in dialyzed patients was tested as a means of improving vitamin K status. Indeed, short-term supplementation with menaquinone-7 (vitamin K2) was found to reduce dp-ucMGP levels in haemodialysis patients [32]. It remains to be seen whether vitamin K supplementation could have an impact on VC in patients with diabetes (through evaluation in large clinical trials).

The limitation of the present study included the small sample size, the lack of evaluation of vitamin K intake and serum vitamin K levels, and the evaluation of marker levels at a single time point. The study would have been strengthened by the presence of a control group of participants with vascular calcification data, so that they could have been compared with diabetic patients. In contrast, one of the study’s main strengths relates to the fact that this was the first study to concomitant evaluate dp-ucMGP/t-ucMGP and peripheral artery calcification in patients with type 2 diabetes.

## Conclusion

In patients with type 2 diabetes, with high cardiovascular risk and normal or slightly altered kidney function, elevated dp-ucMGP levels are independently correlated with the severity of peripheral artery calcification. Hence, dp-ucMGP may be a valuable biomarker in patients with diabetes. The reversibility of the elevation of dp-ucMGP levels and the latter’s relationship with clinical events merit further investigation.

## Abbreviations

MGP: Matrix gla protein; Dp-ucMGP: Dephospho-uncarboxylated matrix gla protein; t-ucMGP: Total uncarboxylated matrix gla protein; p-ucMGP: Phosphorylated uncarboxylated matrix gla protein; c-MGP: Carboxylated matrix gla protein; VC: Vascular calcification; PAD: Peripheral artery disease; CKD: Chronic kidney disease; eGFR: Estimated glomerular filtration rate; HbA1c: Haemoglobin A_1C_; GFR MDRD: Glomerular filtration rate calculated with the modification of diet in renal disease formula; PTH: Parathyroid hormone; CRP: C-reactive protein; CT: Computed tomography; VKA: Vitamin k antagonist; CVD: Cardiovascular disease; SBP: Systolic blood pressure; DBP: Diastolic blood pressure.

## Competing interests

The authors declare that they have no competing interests.

## Authors’ contributions

OB, AH, CEA had substantial contributions to conception and study design and acquisition of data. CVB, ET, EM, MB, RM, SL and ZAM had a substantial contributions to analysis and interpretation of data. SL and ZAM drafted the article. The other authors revised it critically for important intellectual content. All the authors give their final approval of the version to be published.
