# Erratum: Vascular calcification in patients with type 2 diabetes: the involvement of matrix Gla protein

**DOI:** 10.1186/s12933-014-0164-1

**Published:** 2015-01-24

**Authors:** Sophie Liabeuf, Olivier Bourron, Cees Vemeer, Elke Theuwissen, Elke Magdeleyns, Carole Elodie Aubert, Michel Brazier, Romuald Mentaverri, Agnes Hartemann, Ziad A Massy

**Affiliations:** INSERM U1088, Jules Verne University of Picardy, F-80000 Amiens, France; Clinical Research Centre, Division of Clinical Pharmacology, Amiens University Hospital and the Jules Verne University of Picardy, F-80000 Amiens, France; Diabetology Department, AP-HP, Pitie-Salpétrière Hospital and Pierre and Marie Curie University of Paris, F-75005 Paris, France; VitaK, Maastricht University, Maastricht, The Netherlands; Division of Nephrology, Ambroise Paré Hospital, F-92104 Boulogne-Billancourt, France

## Corrections

Following publication of our article [1], it has come to our attention that Olivier Bourron’s name was displayed incorrectly. We publish this correction to update the author list, which is as follows:

Sophie Liabeuf ^1,2^, Olivier Bourron ^3^, Cees Vemeer ^4^, Elke Theuwissen ^4^, Elke Magdeleyns ^4^, Carole Elodie Aubert ^3^, Michel Brazier ^1^, Romuald Mentaverri ^1^, Agnes Hartemann ^3^, and Ziad A. Massy ^1,5^

We apologize for any inconvenience this has caused.
